# Women in acute forensic psychiatric care: comparison of clinical, sociodemographic, and detention-related characteristics in pretrial detention, sentence execution, and court-ordered treatment

**DOI:** 10.1186/s12888-024-05546-0

**Published:** 2024-02-02

**Authors:** Isabella D’Orta, Kerstin Weber, François R. Herrmann, Panteleimon Giannakopoulos

**Affiliations:** 1 Division of Institutional Measures, Medical Direction, Geneva University Hospitals, Geneva, Switzerland; 2 Department of Rehabilitation and Geriatrics, Geneva University Hospitals and University of Geneva, Geneva, Switzerland; 3 Department of Psychiatry, University of Geneva, Geneva, Switzerland; 4 Institute of Global Health, University of Geneva, Geneva, Switzerland

**Keywords:** Women, Prison, Psychiatry, Psychosis court-ordered treatments and women

## Abstract

Compared to men inmates, women display decreased prevalence of severe mental disorder but increased occurrence of substance use disorders (SUD) and higher rates of previous contacts with mental health services. The group of women in detention is highly heterogeneous according to the status of incarceration (pre-trial detention (PTD), sentence execution (SE) and court ordered treatments (COT)). Studies focusing on the comparison of sociodemographic patterns, detention-related and clinical variables between these groups are still lacking. We explored these parameters in 136 women admitted for acute psychiatric care in the sole Geneva forensic unit during a nine year period (2014–2023). Sociodemographic and detention-related data included age, nationality, marital status, presence of children, education attainment, most frequently speaking language, social support, employment before conviction and type of offenses. Clinical variables included the main ICD-10 diagnosis, presence of concomitant SUD, type of personality disorders, presence of suicidal thoughts and attempts at admission, as well as number and mean duration of stays. PTD and SE women had at least 9 years of formal education in 38.9% and 30.3% of cases. Most women in PTD (77.7%), SE (56.6%) and COT (56.2%) groups were Swiss or European citizens. The level of French knowledge was excellent in most of the cases. 43.8% of COT women had at least one child and this percentage is even higher for PTD and SE cases. The employment rate before conviction was also quite high, mainly for PTD and SE (61.1% and 60.6%) and, in a lesser degree, for COT (43.8%) women. Significant social support was present in the vast majority of women without any significant group difference. The distribution of type of offenses did not differ between the three types of detention with a predominance of physical violence, and drug trafficking. The number of stays during the period of reference was significantly higher in COT compared to both SE and PTD women. History of previous inpatient care was also significantly more frequent in COT that SE and PTD women. Adjustment and affective disorders were more often found in SE and PTD cases, these diagnoses were absent in the COT group. In contrast, a main diagnosis of psychotic disorders was found in 62.5% of COT cases compared to only 21.2% in SE and 24.1% in PTD cases. The number of stays, history of inpatient care and diagnosis of psychosis were independent predictors of COT status. In conclusion, the present data reveal the good social integration and emotional support of women needing acute psychiatric care in prison independently of the type of detention. Clinically, women in PTD and SE display more often emotional distress whereas those in COT suffer from acute psychotic symptoms with previous history of psychiatric care and multiple inpatient stays.

## Introduction

Women represent 5% of the European prison population and are likely to have specific healthcare needs that remain poorly explored [1–3]. The psychosocial risks of female offenders include childhood victimization, extreme poverty and educational problems but also severe mental disorder, substance use disorder (SUD), depression and anxiety [4]. Several lines of evidence indicated that women in prison suffer from more frequent mental health problems than men, and are of higher risk of suicide and overdose death following release [5–12]. The overall increase of psychiatric morbidity in prisons in last decades is thought to be even more pronounced among women [13]. Major depression, anxiety disorders and SUD seems to be the most prevalent disorders among pre-trial and sentenced women [3, 14–17]. Compared to men detainees, women display decreased prevalence of severe mental disorder associated with personality disorders [18] but an increased occurrence of SUD [3, 15, 16] and higher rates of previous contacts with mental health services because of a pre-existing diagnosis [12].

Whether or not referral to forensic psychiatry reduces the gender differences in psychiatric morbidities is still matter of debate. Among forensic patients and in respect to socio-demographic and criminological characteristics, no gender-related differences were found in age at incarceration, type of employment prior to detention and previous psychiatric treatment [19]. However, women were more likely than men to have committed homicide and arson, display aggressive behaviour during treatment, and have an history of sexual abuse [6]. Compared to men, women were more likely to receive longer prison terms and higher antipsychotic dosages in cases with schizophrenia, but are less frequently assigned to court-ordered treatments (COT) for addiction [20, 21].

Studies focusing on the characteristics of women inmates needing acute psychiatric care are rare. In an early study on forensic psychiatric care in the German-speaking part of Switzerland, Krammer and colleagues [22] reported that 2/3 of women were not in a stable relationship, more than half did not complete a school degree, and 3/4 had no employment prior to detention. The predominance of SUD and drug-related crimes characterized this population [22]. Women treated in forensic settings displayed the same level of psychopathy independently of the type of detention [24]. Not surprisingly, a diagnosis of schizophrenia was the main determinant of COT [25]. Women referred to forensic outpatient services displayed more often psychotic disorders with comorbid SUD [23] whereas personality disorders were more often diagnosed in inpatient settings [17].

One main difficulty for developing gender-specific policies resides to the variability of care needs according to the status of incarceration. Pre-trial detention (PTD), SE and COT correspond to three radically different conditions that impact on care needs both in men and women [24, 25]. The overuse of PTD worldwide has created deleterious conditions in detention facilities exposing large numbers of people to health risks [26, 27]. In contrast to SE, there are significant gender-related differences in criminal recidivism in juveniles entering pretrial detention: age at first incarceration and oppositional defiant disorder determines increased risk for recidivism in boys whereas generalized anxiety disorder predicts reoffending in girls [28]. Moreover and compared to SE, PTD is associated with moderate to strong adherence to medical care [29]. COT are reserved to cases of legal insanity based on cognitive and/or volitional impairment according to countries. There is a general agreement that a diagnosis of schizophrenia indicates a lack of accountability, whereas opinions differ among legal frameworks regarding personality disorders, psychopathy, and substance use disorders [30]. COT take usually place in high and medium-security hospitals [31, 32] and raise ethical questions, as length of stay may be long and often indefinite. Psychiatric care in secure prison-based settings is thus restrictive and of high cost for both men and women [33, 34].

The Swiss Penal Law distinguishes between penalties corresponding to SE and COT, referred to as therapeutic measures. The latter are ordered when SE alone is not sufficient to counterbalance the risk of future offending. Therapeutic measures can be pronounced in conjunction with a custodial sentence, or against offenders who are criminally irresponsible and cannot be sentenced to a penalty. The decision of the Court is based on a psychiatric expert assessment that provides an opinion on the prospect of success of the treatment, the probability of future offences, and the ways in which the measure may be implemented. COT are reviewed annually according to the best interest of both the individual and the public safety, because their duration can far exceed the sentence related to the seriousness of the crime, which typically determines the duration of imprisonment in SE.

To date, there are no available data about the gender-related differences in care needs between these three types of detention. However, this information may be crucial when designing the models of psychiatric care for women detainees. In one previous study of 200 detainees admitted in an acute care psychiatric unit, we reported an overrepresentation of personality and psychotic disorders among COT cases [35]. However, this sample included only 27 women without distinction between SE and PTD and after exclusion of SUD cases. In this study, we explore the socio-demographic, detention-related and clinical characteristics of 136 women admitted for acute psychiatric care in the sole Geneva forensic unit during a nine year period. Our main hypothesis is that in contrast to the observations made in male-predominant samples, women in forensic care would display better social integration and support independently of the type of detention. Using regression models, we also aimed to identify whether sociodemographic, clinical and detention-related variables could predict the COT status (as compared to the PTD and SE status) taking into account their interdependence. Our hypothesis was that the presence of severe psychiatric disorder and history of psychiatric care previous to conviction would be associated with COT whereas acute and transitory depressive reactions would characterize PTD.

## Materials and methods

### Study sample and data collection

We examined retrospectively the psychiatric records corresponding to all admissions of women during a nine-year period (2014–2023) in UHPP (Unité hospitalière de psychiatrie penitentiaire), a unique ward of 15 beds specially designed for acute psychiatric care of prison detainees from the French speaking counties in Switzerland. This unit is located in a medium-security hospital (referred to as Curabilis) that is also in charge of the COT for French -speaking offenders in Switzerland. Women were admitted to this ward under three possible status: PTD, SE and COT. The total mean number of admissions per year for the period of reference was of 261. This number corresponds to the total number of annual admissions. Since the study focuses exclusively on women, the only inclusion criterion has been female gender. It must be noted that no transgender female patient has been admitted during the period of the study, so the sample includes uniquely cisgender female patients.

Importantly, there is no crisis discharge in this unit since psychiatric admissions cannot be refuted and number of beds is usually sufficient to cover the needs of acute care. In the rare cases of bed lacking, the hospital stays take place in a general psychiatry unit. Patients are admitted to the UHPP in the presence of acute symptoms associated with self or others-threatening behaviour and need for urgent psychiatric care. All of the patients were admitted based on the recommendation of the psychiatrist who assumes the care process in inpatient (COT in Curabilis) and outpatient (PTD and SE in regular prison) settings. The final sample included 136 women (mean age: 38.9 ± 16.2, age range: 20–63). Among them, there were 54 PTD, 66 SE and 16 COT cases (art 59, 60 Swiss Criminal Code). Every patient was assigned an identification number that was derived from the name and birth date and subsequently encrypted. Sociodemographic data included age, nationality, marital status (at initial admission), children (binary), education attainment, most frequently speaking language (French or other), social support (binary), type of employment before conviction and type of offenses. Fluent French was assessed in an empiric way, observing and evaluating the abilities of the subject to understand and to communicate in the context of the ward. As a rule, an interpreter is not needed if the patient is able to understand basic information related to their stay in the hospital and communicate effectively during clinical encounters. The patient was asked to describe in details her symptoms and comment on their evolution during the hospitalization. In that case, the level of French was considered fluent. Social support was determined by the presence of relevant ones assisting the detainee, visiting her, or providing her with basic material support (such as clothes, comfort food, tobacco etc.). It can include family members, friends or any other person with an affective relationship with the detainee. Financial support provided by the government was not considered as social support.

According to the Swiss Penal Law, the main type of offenses are classified as follows: physical violence, property violation, drug trafficking, financial crimes (including unpaid fines, fraud), threat, sequestration and kidnapping (including stalking), violence against the forces of the order, honour and privacy, sexual offenses, road traffic offenses, arson, illegal immigration and gun law violation. Among clinical variables, psychiatric outpatient and inpatient history before conviction, main ICD-10 diagnosis, presence of substance use disorders (SUD), types of personality disorders, suicidal attempt and thoughts (during the period of reference), mean length and total number of stays during the period of reference were recorded. All of the ICD-10 clinical diagnoses were made prospectively by two independent, board-certified psychiatrists (prior and during the hospital stay), blind to the scope of the study. Only cases with concordant psychiatric diagnoses were considered in this sample.

### Statistical analysis

Fisher exact, unpaired Student t and Mann-Whitney u tests were used to compare sociodemographic and clinical variables according to the time of detention. Age and length of stays were treated as quantitative variables. Nationality (EU, extra EU, Swiss), marital status (married, separated-divorced, single), education (drop-out, obligatory, high school and university, apprenticeship), employment before conviction, type of offenses reported in the present sample (unpaid fine, property violation, fraud, arson, physical violence, drug trafficking, honor and privacy, stalking, other), previous outpatient and inpatient care, number of stays (1, 2–10, > 10) were treated as ordinal variables. Fluent French, social support, children, SUD, suicidal thoughts and attempts were treated as binary variables. Psychiatric diagnoses included adjustment disorders, bipolar and depressive disorders (ICD-10 codes F32-33), personality disorders (F60), and psychosis (ICD-10 codes F20-F29). The distribution of personality disorders included the most frequently occurred borderline, narcissistic and histrionic types, Cluster A (one schizoid and one paranoid), and not otherwise specified. The significance level was set at *P* < 0.05 but was corrected to *P* < 0.00625 for multiple testing by using the Benjamini-Hochberg method [36]. Univariate and multiple logistic regression models that included only the variables with significant group differences were developed to assess the determinants of COT versus SE and PTD taking into account the interdependence of the variables of interest. This method is complementary to group comparisons and makes possible to define the relative weight of each independent variable retained into the model in the prediction of the dependent variable (COT status). All statistical analysis were performed using Stata 17.0.

## Results

Group comparisons between PTD, SE and COT women are illustrated in Table 1. Socio-demographic factors as well as type of offense were comparable among the three groups. PTD and SE women had at least 9 years of formal education in 38.9% and 30.3% of cases. Most women in PTD (77.7%), SE (56.6%) and COT (56.2%) needing acute psychiatric care were Swiss or European citizens. The level of French knowledge was excellent in most of the cases. 43.8% of COT women had at least one child and this percentage is even higher for PTD and SE cases. The employment rate before conviction was quite high, mainly for PTD and SE (61.1% and 60.6%) and, in a lesser degree for COT (43.8%). Significant social support was present in the vast majority of women in all three groups. The distribution of type of offenses did not differ between the three types of detention with a clear predominance of physical violence (30.4%), followed by drug trafficking (14.8%) and property violation (13.3%).

**Table 1:** Sociodemographic, detention-related and clinical variables in the present series

	Type of detention								p	BH
	Pre-trial			Sentence		COT		Total		
N	54 (39.7%)			66 (48.5%)		16 (11.8%)		136 (100.0%)		
**Age**	38.9 [16.2]			36.3 [16.2]		35.6 [10.4]		36.6 [15.2]	0.311	
**Nationality**									0.173	
EU	22 (40.7%)			19 (28.8%)		4 (25.0%)		45 (33.1%)		
Extra-EU	12 (22.2%)			28 (42.4%)		7 (43.8%)		47 (34.6%)		
Swiss	20 (37.0%)			19 (28.8%)		5 (31.2%)		44 (32.4%)		
**Marital status**									0.560	
Single	28 (51.9%)			29 (43.9%)		7 (43.8%)		64 (47.1%)		
Separed-divorced-widowed	17 (31.5%)			26 (39.4%)		4 (25.0%)		47 (34.6%)		
Married	9 (16.7%)			11 (16.7%)		5 (31.2%)		25 (18.4%)		
**Children**	29 (53.7%)			38 (57.6%)		7 (43.8%)		74 (54.4%)	0.612	
**Education**									0.489	
Drop-out	16 (29.6%)			25 (37.9%)		6 (37.5%)		47 (34.6%)		
Obligatory schooling	17 (31.5%)			21 (31.8%)		5 (31.2%)		43 (31.6%)		
Apprenticeship	13 (24.1%)			17 (25.8%)		5 (31.2%)		35 (25.7%)		
High school, university	8 (14.8%)			3 (4.5%)		0 (0.0%)		11 (8.1%)		
**Language (French)**	46 (85.2%)			58 (87.9%)		15 (93.8%)		119 (87.5%)	0.762	
**Social support**	46 (85.2%)			62 (93.9%)		14 (87.5%)		122 (89.7%)	0.241	
**Employment before conviction**									0.170	
Invalidity pension	6 (11.1%)			15 (22.7%)		3 (18.8%)		24 (17.6%)		
Students	0 (0.0%)			1 (1.5%)		0 (0.0%)		1 (0.7%)		
None	15 (27.8%)			10 (15.2%)		6 (37.5%)		31 (22.8%)		
Yes	33 (61.1%)			40 (60.6%)		7 (43.8%)		80 (58.8%)		
**Type of offenses**									0.096	
Unpaid fine	3 (5.6%)			4 (6.2%)		0 (0.0%)		7 (5.2%)		
Property violation	6 (11.1%)			11 (16.9%)		1 (6.2%)		18 (13.3%)		
Fraud	2 (3.7%)			7 (10.8%)		0 (0.0%)		9 (6.7%)		
Arson	2 (3.7%)			2 (3.1%)		2 (12.5%)		6 (4.4%)		
Physical violence	13 (24.1%)			20 (30.8%)		8 (50.0%)		41 (30.4%)		
Drug trafficking	8 (14.8%)			12 (18.5%)		0 (0.0%)		20 (14.8%)		
Other	16 (29.6%)			6 (9.2%)		5 (31.2%)		27 (20.0%)		
Honor and privacy	2 (3.7%)			2 (3.1%)		0 (0.0%)		4 (3.0%)		
Stalking	2 (3.7%)			1 (1.5%)		0 (0.0%)		3 (2.2%)		
**Number of stays**	a				b		a,b		0.004	*
1	38 (70.4%)			49 (74.2%)		6 (37.5%)		93 (68.4%)		
2–10	16 (29.6%)			16 (24.2%)		7 (43.8%)		39 (28.7%)		
> 10	0 (0.0%)			1 (1.5%)		3 (18.8%)		4 (2.9%)		
**Length of stay**	18.0 [32.0]			21.5 [31.0]		14.0 [22.0]		18.5 [30.5]	0.544	
**Psychiatric history**	c				d		c,d		< 0.001	*
None	7 (13.0%)			20 (30.3%)		0 (0.0%)		27 (19.9%)		
Outpatient	12 (22.2%)			22 (33.3%)		1 (6.2%)		35 (25.7%)		
Inpatient	35 (64.8%)			24 (36.4%)		15 (93.8%)		74 (54.4%)		
**Main diagnosis (ICD-10)**			e	f			e,f		0.004	*
Adjustment disorder	12 (22.2%)			7 (10.6%)		0 (0.0%)		19 (14.0%)		
Affective disorders (bipolar and depressive)	12 (22.2%)			23 (34.8%)		0 (0.0%)		35 (25.7%)		
Personality disorders	14 (25.9%)			20 (30.3%)		6 (37.5%)		40 (29.4%)		
Psychotic disorders	13 (24.1%)			14 (21.2%)		10 (62.5%)		37 (27.2%)		
Other	3 (5.6%)			2 (3.0%)		0 (0.0%)		5 (3.7%)		
**Substance use disorders**	16 (29.6%)			20 (30.3%)		5 (31.2%)		41 (30.1%)	1.000	
**Personality**									0.576	
None	28 (51.9%)			29 (43.9%)		7 (43.8%)		64 (47.1%)		
Not otherwise specified	4 (7.4%)			7 (10.6%)		1 (6.2%)		12 (8.8%)		
Borderline	20 (37.0%)			25 (37.9%)		6 (37.5%)		51 (37.5%)		
Cluster A	2 (3.7%)			2 (3.0%)		0 (0.0%)		4 (2.9%)		
Narcissistic/Histrionic	0 (0.0%)			3 (4.5%)		2 (12.5%)		5 (3.7%)		
**Suicidal issues**									0.136	
No	26 (48.1%)			28 (42.4%)		10 (62.5%)		64 (47.1%)		
Suicidal attempt	9 (16.7%)			17 (25.8%)		5 (31.2%)		31 (22.8%)		
Suicidal thoughts	19 (35.2%)			21 (31.8%)		1 (6.2%)		41 (30.1%)		

BH Threshold: p = 0.00882

Kruskal Wallis for continuous variables

a: PTD vs COT; p = 0.005

b: SE vs COT; p=0.011

Fisher's exact test for factor variables

c and d: PTD vs COT; p = 0.001

e: PTD vs COT p = 0.007

f: SE vs COT p = 0.002

The distribution of the number of stays was significantly different between the three groups. COT women were much more frequently admitted in UHPP compared to the two other groups. 18.8% of COT women were admitted for more than 10 stays during the fixed time period but this percentage was only of 1.5% for SE and 0% for PTD cases. In the same line, history of inpatient, but not outpatient, care prior to incarceration was significantly more frequent in COT (93.8%), compared to both PTD (64.8%) and SE (36.4%) cases. The distribution of main diagnoses was also strikingly different between the three groups. A predominance of adjustment and affective disorders was found in SE and PTD cases, these diagnoses were absent in COT group. In contrast, a main diagnosis of psychotic disorders was found in 62.5% of COT cases compared to only 21.2% in SE and 24.1% in PTD cases. Of importance, the occurrence of personality disorders and SUD did not differ between the three groups. These differences were highly significant and persisted after Benjamini-Hochberg correction for multiple comparisons.

Among the variables included in group comparisons, the number of stays, history of inpatient care and diagnosis of psychosis were significantly associated with COT status in univariate logistic regression models. To take into account the interdependence of some independent variables, multivariable models were also considered. The increase of the number of stays was clearly related to the COT status in women in multivariable models with an OR of 49.5 for more than 10 stays. Both the history of inpatient care and presence of psychotic disorders were independent determinants of the COT status (Table 2).

**Table 2:** Univariate (unadjusted OR) and multiple (adjusted OR) logistic regression associated with the type of detention

	Unadjusted		Adjusted	
Characteristics	Odds Ratio	P	Odds Ratio	P
Number of stays				
1	1.00 (1.00,1.00)		1.00 (1.00,1.00)	
2–10	3.17 (0.99,10.15)	0.0518	3.68 (1.07,12.67)	0.0391
> 10	43.50 (3.91,484.15)	0.0021	49.50 (3.64,673.39)	0.0034
**PIC**	12.55 (2.73,29.23)	0.0012	9.71 (2.92,20.95)	0.0016
**Psychotic disorders**	5.74 (1.91,17.23)	0.0018	6.50 (1.92,21.95)	0.0026

PIC: previous inpatient care

## Discussion

The present data reveal the good social integration and emotional support of women needing acute psychiatric care in prison. Their clinical profiles vary as a function of the type of detention. Women in PTD and SE display more often emotional distress. COT detainees represent a distinct subgroup with increased needs for mental health care as documented by heavy use of acute psychiatric wards, history of inpatient care prior to incarceration and presence of chronic psychotic disorder.

One should keep in mind that in the Swiss law, COT are proposed by a psychiatric expert only when there is reasonable chance to reduce recidivism. As frequently indicated in previous studies, young ethnic-minority male patients with low education are prone to negative assumptions about their potential to evolve positively in medium-security hospitals that may, in fact, preclude the proposal of COT [37–39]. Our data suggest that this could not be the case for women. Of importance, PTD and SE women had at least 9 years of formal education in 38.9% and 30.3% of cases respectively. These percentages are comparable to those reported by Krammer and colleagues [22] in female prisoners under forensic psychiatric care and even much higher to those usually reported for male inmates referred to inpatient psychiatric care [35, 40]. In the same line, most women in our sample were Swiss or European citizens speaking fluently French in the vast majority of cases and benefit from significant social support. This demographic profile contrasts sharply with previous reports pointing to the frequent presence of foreign national male prisoners with acculturation problems among detainees needing psychiatric care [41–43]. The presence of significant social support in more than 85% of women independently of their detention status also contrasts with the paucity or absence of such support reported both in depressed male-predominant samples of detainees in regular prison and secure forensic hospitals [44, 45]. The same demographic characteristics were found in COT women who were mostly admitted for personality disorders and psychosis supporting the idea that, at least in the Swiss prison system, women needing psychiatric care are not exposed to major acculturation problems.

Some other demographic and detention-related characteristics merit further comments. Unlike the observations made in male-predominant cohorts [35, 46], we found no association between single marital status and COT in our sample. In the same line, 43.8% of them were mother and this percentage reaches more than 50% in PTD and SE cases. A similar pattern was found in respect to the unemployment rate before conviction that was of 15.2% in SE, 27.8% in PTD and did not exceed 37.5% in COT cases. These data parallel previous gender comparisons in forensic patients further supporting the idea of a good social integration and emotional support for incarcerated women needing acute psychiatric care [19, 47]. However, the proportion of married women (18.4%) was comparable to that reported in forensic male samples [35] indicating that the majority of women detainees needing psychiatric care were not in a stable relationship prior to detention [22]. The relative overrepresentation of violent and drug crimes in the present sample is also consistent with previous reports that pointed to the violent nature of offenses and frequent occurrence of drug-related convictions in women under psychiatric care [6, 47, 48]. Although the percentage of physical violence reached 50% in COT women compared to 30.8% for SE and 24.1% for PTD suggesting an increased tendency for violent acting out in the former group, this difference did not reach statistical significance possibly because of sample limitation.

Importantly, our study reveals the clinical characteristics of women addressed in acute forensic settings. As one could expect, history of psychiatric inpatient care prior to conviction was the rule among women with COT. This reflects a severe and long-standing vulnerability to mental disorders in this population, an evidence already reported for men and in mixed samples [35]. COT status is associated with a significant increase in the number of referrals for crisis interventions [49]. Almost 19% of COT women were admitted more than 10 times in UHPP compared to 1.5% in SE and none in PTD groups. This difference was highly significant and persisted after correction for multiple comparisons as also reported in mixed samples [35, 49]. In general psychiatry, the revolving door phenomenon was associated with drug addiction, young age, low level of education, single status but also mood or psychotic disorders [50–53]. Among these factors, the presence of psychotic disorders was the only that could explain our findings since this diagnosis is overrepresented among COT women. However, our multivariable analysis indicate that the association between COT status and increased use of acute psychiatric beds persists after controlling for the effect of this diagnosis and previous inpatient care. In line with the high frequency of episodes of inpatient care before conviction, COT women may be characterized by a long-lasting vulnerability to acute psychiatric episodes despite their intensive care program in medium-security hospitals. Alternatively, the COT status could also be associated with increased use of compulsory admissions to acute psychiatric wards in order to discharge the teams of Curabilis that have to face severe behavioral disturbances.

The distribution of clinical diagnosis was radically different between COT and the other two groups of women. Adjustment and affective disorders occurred more frequently in PTD and SE compared to COT groups pointing to the emotional distress of women not only at the pre-trial period but also during SE [42, 54–56]. In previous studies, this distress was associated with increased vulnerability to suicide among SE women. In line with this idea, when one considers suicidal issues as a binary variable, they were present in almost 52% of PTD and 57.6% of SE women in the present sample. By comparison, this prevalence was of 48.2% in a male-predominant PTD and SE sample admitted to acute psychiatry wards [35]. Psychotic disorders were clearly more frequent among COT women needing acute psychiatric care with affecting 62.5% of the cases. The strong association between psychosis and COT in women survived after correction for multiple comparisons and remained present in adjusted multivariable regression models. Similar data were reported by Ribeiro and colleagues [57] in a medium security unit but also by Collier and Friedman [23] in a sample of women prisoners referred to the forensic psychiatry service. Several studies in male-exclusive or predominant samples also showed that schizophrenia is one of the main determinants of COT in most Western countries [34, 58–61]. In contrast to previous reports in male inmates [34, 35], personality disorders were equally distributed across the three types of detention in women. It is likely that for the subsample of COT women included in this study, the referral to forensic inpatient services was mostly related to the severity of their psychotic symptoms and not the dramatic expression of their personality disorder. A last point to consider is the relatively modest percentage of women with SUD in the present sample. The rate of occurrence of these conditions were close to 30% without any difference related to the detention status. Previous studies in women sample led to variable percentages of SUD diagnosis in European countries depending on the reference to regular prison or forensic settings. In regular prisons, SUD prevalence in women were consistently higher that in men and reached 50 to 60% [3, 15] without comorbid personality disorders and severe mental disorder. In forensic settings this percentage was systematically lower [16, 17, 57]. In the present sample, SUD was consistently associated with another psychiatric morbidity pointing to the fact that the psychological vulnerability of women in forensic care cannot be exclusively attributed to drug abuse.

Last but not least, the present data should be interpreted in the light of traditional gender-related roles. Stereotypes about women are frequent and encompass a wide range of expectations related to behaviors, roles and social codes. Gender stereotypes have a major role in socioeconomic disparities, access to education and mental health. Moreover, they can also influence the way women are considered in prison, maintaining unfair assumptions about their behaviors and needs. Addressing and challenging gender stereotypes is crucial to promote diversity in prison. The role of women in the society, their commitment to training and education and expectations regarding motherhood are a few of these themes. Our data show that in our setting, women needing psychiatric care have a good level of education, dismantling the idea that women in prison are mainly uneducated and with poor professional vocation. The risk of this stereotype is to consider that women detainees are less prone to commit in educational or professional training, supporting them in a less efficient way towards a professional path and being less empowering. This study also highlights the frequency of mothers undergoing a COT. Stereotypes about motherhood are widespread and they may lead, for instance, to disproportionate charge of expectations towards patients and the way they embrace their experience of parenthood.

## Strengths and limitations

Strengths of the present study includes the presence of a single unit of acute psychiatric care in prison that decreases the variability in the criteria of admission, and use of multivariable models that make it possible to control for the interdependence between the clinical and demographic variables. Several limitations should, however, be considered. First and given the results obtained in our previous studies in male-predominant samples [29, 42], the present study focuses on the acute forensic care of women detainees without the inclusion of a control group of male inmates. Second, one could speculate that the increased number of stays in COT cases reflects the longer duration of sentence. However, this is an unlikely scenario since the number of stays considered was not fixed in respect to the total duration of sentence for each case but in respect to an a priori fixed period of reference (9 years). Of note, the distribution of the number of stays was quasi-identical in PTD and SE cases despite the fact that the latter are usually convicted to long periods of incarceration. As usually, the number of COT women remains limited. To be close to a real-life situation, clinical diagnosis was made using two independent clinicians blinded to the scope of the study but without use of standardized diagnostic questionnaires. Only COT patients needing acute psychiatric care were considered leading to an overrepresentation of unstable cases that did not cover the full spectrum of this type of treatment. Binary data on usual language may mask more complex realities in respect to the ethnic and cultural background of the women in all three groups. Future studies in larger samples including COT patients without acute care needs, standardized assessment of clinical diagnosis and demographic factors, are warranted to explore gender-related specificities as a function of the type of detention and design appropriate care intervention for women.

## Conclusions

In conclusion, the present data reveal the good social integration and emotional support of women needing acute psychiatric care in prison independently of the type of detention. This subgroup is also less exposed to SUD compared to regular inmates. In terms of clinical diagnosis, there was a clear distinction between the emotional distress observed in PTD and SE and the predominance of psychotic symptoms in COT women. These latter show a very frequent referral to inpatient care before conviction. These observations point to the need for implementing distinct mental health screening procedures for women as a function of the detention type. In particular, tools related to the detection of acute depressive reactions should be incorporated in the routine assessment of PTD and SE women whereas care strategies in COT should include careful documentation of psychotic symptoms.

## Data Availability

Data and Material are available upon request from corresponding author. The raw data supporting the conclusions of this article will be made available by the corresponding author, without undue reservation.
